# Statistical modeling and optimization of culture conditions by response surface methodology for 2,4- and 2,6-dinitrotoluene biodegradation using *Rhodococcus pyridinivorans* NT2

**DOI:** 10.1007/s13205-016-0468-9

**Published:** 2016-07-19

**Authors:** Debasree Kundu, Chinmay Hazra, Ambalal Chaudhari

**Affiliations:** School of Life Sciences, North Maharashtra University, Jalgaon, 425 001 Maharashtra India

**Keywords:** *Rhodococcus*, Biodegradation, Dinitrotoluene, Medium development, Optimization

## Abstract

To improve biodegradability (% biodegradation) and specific growth rate of *Rhodococcus pyridinivorans* NT2, culture medium and environmental parameters were screened and optimized using the statistical design techniques of Plackett–Burman and response surface methodology. Of the process variables screened, DNTs (2,4-DNT and 2,6-DNT), MgSO_4_·7H_2_O, temperature and inoculum size (O.D.) were selected as the most important (*P* value <0.05) factors. In multiresponse analysis of central composite design, medium formulation consisting of 474/470 mg l^−1^ 2,4-DNT/2,6-DNT, 0.11 g l^−1^ MgSO_4_·7H_2_O, 37.5 °C temperature and 1.05 OD inoculum size were found to predict maximum % degradation and specific growth rate of 97.55 % and 0.19 h^−1^, respectively. The validity of the optimized variables was verified in shake flasks. The optimized media significantly shortened the time required for biodegradation of DNTs while providing a nearly 30 % (for 2,4-DNT) and 70 % (for 2,6-DNT) increased biodegradation along with 5.64-fold increase in specific growth rate for both DNTs.

**Electronic supplementary material:**

The online version of this article (doi:10.1007/s13205-016-0468-9) contains supplementary material, which is available to authorized users.

## Introduction

2,4-Dinitrotoluene (2,4-DNT) and 2,6-dinitrotoluene (2,6-DNT) are the most common isomers produced during 2,4,6-trinitrotoluene (TNT) synthesis (Nishino et al. 2000) and are used commercially as an intermediate in the production of herbicides, dyes, and synthetic foams (Spain 1995; Mulla et al. 2014). Besides, DNTs are used as waterproofing, plasticizing and gelatinizing agent in explosives and as a modifier for smokeless powders (Kuşçu and Sponza 2011). While DNTs can be mineralized by many microorganisms isolated from the DNT-contaminated sites under laboratory conditions, DNT’s long-term persistence in the contaminated soil and groundwater is often observed (Han et al. 2011). Thus, the environmental impact of exposure of DNTs is a major public concern and is regulated by the US Environmental Protection Agency (EPA) as priority pollutants (Han et al. 2011; Hudcova et al. 2011; Mulla et al. 2014).

To date, numerous studies reported the remediation of TNT and DNTs in contaminated lands, including ex situ and in situ techniques (Das et al. 2013; Gumuscu and Tekinay 2013). Also, the metabolic fate, toxicity, and biotransformation pathways in fungi, bacteria and plants have already been the subject of several studies (Bradley et al. 1994; Spain 1995; Nishino et al. 2000). Current research has mainly focused on the isolation of various microbes to mineralize/degrade nitroaromatics aerobically or anaerobically, and deciphering the catabolic pathways and enzymes involved in degradation (Ye et al. 2004; Kulkarni and Chaudhari 2007; Ju and Parales 2010; Mulla et al. 2011a, b, 2013; Singh et al. 2014). Although the DNT concentrations used in these studies were much higher than would be needed for successful applications of biological treatment in environmental systems, there is no reported instances where DNT biodegradation studies were conducted beyond 50 µM to 1 mM.

We have recently isolated and characterized a *Rhodococcus pyridinivorans* NT2 from a pesticide-manufacturing facility that can degrade 4-NT (Kundu et al. 2013), 2,4-DNT (Kundu et al. 2015a) as well as 2,6-DNT (Kundu et al. 2015b). As a continuation of our research on the microbial treatment of nitrotoluene-contaminated wastewater, it is imperative to formulate an optimized cultivation strategy for enhanced degradation of DNTs to develop more efficient methods of treating such wastewater. Incidentally, to our knowledge, information regarding the multivariate statistical optimization in biodegradation of DNTs is not available. Therefore, the objective of this work was to increase the biodegradability (% biodegradation) of 2,4-DNT and 2,6-DNT using *R. pyridinivorans* NT2 by optimizing the media ingredients and environmental parameters. For this purpose, mathematical modeling and statistical analysis using Plackett–Burman design (PBD) and response surface methodology (RSM) were used to evaluate the main and interactive effects of process parameters on the biodegradation of DNTs and to determine their optimal values. Furthermore, a central composite design (CCD) model was developed to predict the removal of these isomers. Finally, numerical optimization based on desirability function was carried out to verify and validate the process.

## Materials and methods

### Chemicals

2,4-DNT (97 %) and 2,6-DNT (98 %) were obtained from Sigma-Aldrich (St. Louis, MO, USA). All inorganic chemicals were of analytical grade unless specified otherwise and were obtained from Hi-Media, Mumbai (India).

### Microorganism and culture medium

The actinobacterium *R. pyridinivorans* NT2 was used in this study (Kundu et al. 2013, 2015a). A basic mineral salt medium (MSB) (pH 7.0 ± 0.2) containing 0.75 g l^−1^ dipotassium hydrogen phosphate (K_2_HPO_4_), 0.2 g l^−1^ potassium dihydrogen phosphate (KH_2_PO_4_), and 0.09 g l^−1^ magnesium sulfate (MgSO_4_·7H_2_O) was used to grow the microorganism. Solid media were prepared by the addition of 15 g l^−1^ agar to the MSB medium. Prior to use, the media were sterilized in an autoclave at 121 °C for 15 min. The growth media were prepared by adding 2,4-DNT or 2,6-DNT of the required concentration (from filter-sterilized stock solutions in acetone) to the MSB. Acetone was removed by evaporation prior to the addition of the aqueous medium. Seed culture of the isolate was prepared with 18-h-old inoculum (OD_600_ = 0.5; ~1.0 × 10^7^ CFU ml^−1^; ~1.6 mg CDW l^−1^) in the MSB. The pure culture was maintained by subculturing every 45 days in a 250-ml conical flask containing 100 ml of MSB and 2,4-DNT/2,6-DNT at an initial concentration of 100 mg l^−1^. All biodegradation experiments were performed in 500-ml conical flask containing 100 ml of media, as per the statistical design given in Supplementary Table 1.

### Identification of important variables and range finding by PBD

The first screening step in optimization is to identify the variables which have significant effects on system response(s). For this, a two-level 12-run PBD in duplicate (Plackett and Burman 1946; Khuri and Mukhopadhyay 2010) was used to screen nine variables viz. 2,4-DNT/2,6-DNT, K_2_HPO_4_, KH_2_PO_4_, MgSO_4_·7H_2_O, yeast extract, pH, temperature, agitation and inoculum size at high (+1) and low (−1) levels (Table 1) to maximize the responses (% degradation and specific growth rate). In the 12-run PBD, each row represents an experiment and each column represents an independent variable (Tables 1a, 2b for 2,4- and 2,6-DNT, respectively).

**Table 1:** PBD matrix adopted in the screening study for biodegradation of (a) 2,4-DNT and (b) 2,6-DNT

(a) Run no.	Experimental variables									Response 1	Response 2
	2,4-DNT (mg l^−1^)	MgSO_4_·7H_2_O (g l^−1^)	K_2_HPO_4_ (g l^−1^)	KH_2_PO_4_ (g l^−1^)	Yeast extract (g l^−1^)	Temperature (°C)	pH	Agitation (rpm)	Inoculum (OD)	Specific growth rate (h^−1^)	Biodegradability (% biodegradation)
1	400	0.05	2	2	1	15	5	110	1	0.11	38
2	100	0.1	0.05	2	1	15	9	200	1	0.19	94
3	100	0.05	2	0.05	1	40	5	200	1	0.14	72
4	100	0.1	2	0.05	1	40	9	110	1	0.14	45
5	100	0.1	2	2	0.1	15	5	200	0.1	0.1	56
6	100	0.05	0.05	0.05	0.1	15	5	110	0.1	0.06	65
7	400	0.1	0.05	0.05	0.1	40	5	200	0.1	0.13	89
8	400	0.05	2	2	0.1	40	9	200	0.1	0.08	60
9	100	0.05	0.05	2	0.1	40	9	110	0.1	0.16	80
10	400	0.1	0.05	2	1	40	5	110	1	0.104	73
11	400	0.1	2	0.05	0.1	15	9	110	0.1	0.11	45
12	400	0.05	0.05	0.05	1	15	9	200	1	0.06	81

(b) Run no.	Experimental variables									Response 1	Response 2
	2,6-DNT (mg l^−1^)	MgSO_4_·7H_2_O (g l^−1^)	K_2_HPO_4_ (g l^−1^)	KH_2_PO_4_ (g l^−1^)	Yeast extract (g l^−1^)	Temperature (°C)	pH	Agitation (rpm)	Inoculum (OD)	Specific growth rate (h^−1^)	Biodegradability (% biodegradation)
1	300	0.05	2	2	1	15	5	110	1	0.1	78
2	300	0.1	0.05	2	1	40	5	110	0.1	0.11	92
3	100	0.05	2	0.05	1	40	5	200	1	0.14	43
4	100	0.1	2	0.05	1	40	9	110	0.1	0.14	35
5	100	0.1	0.05	2	1	15	9	200	1	0.17	63
6	100	0.05	0.05	0.05	0.1	15	5	110	0.1	0.07	70
7	300	0.1	0.05	0.05	0.1	40	5	200	1	0.12	54
8	300	0.05	0.05	0.05	1	15	9	200	0.1	0.04	70
9	300	0.05	2	2	0.1	40	9	200	0.1	0.07	80
10	100	0.05	0.05	2	0.1	40	9	110	1	0.15	60
11	100	0.1	2	2	0.1	15	5	200	0.1	0.1	42
12	300	0.1	2	0.05	0.1	15	9	110	1	0.1	90

**Table 2:** Experimental design and results of CCD of response surface methodology for the optimization of biodegradation of (a) 2,4-DNT and (b) 2,6-DNT

(a) Exp. run no.	Experimental variables				Specific growth rate (h^−1^)		Biodegradability (% biodegradation)	
	2,4-DNT (X_1_)	MgSO_4_·7H_2_O (X_2_)	Temperature (X_3_)	Inoculum size (X_4_)	Exp.	Pred.	Exp.	Pred.
1	600	0.08	50	0.5	0.17	0.12	86	82
2	600	0.15	30	1.5	0.11	0.14	80	81
3	300	0.15	30	1.5	0.18	0.13	78	80
4	300	0.08	50	1.5	0.17	0.11	63	62
5	600	0.08	30	0.5	0.15	0.16	32	41
6	300	0.15	30	0.5	0.03	0.1	80	84
7	300	0.08	30	0.5	0.08	0.12	88	89
8	450	0.115	40	1	0.18	0.16	79	82
9	450	0.115	40	1	0.16	0.15	81	77
10	300	0.08	30	1.5	0.1	0.13	70	67
11	300	0.15	50	1.5	0.16	0.12	60	65
12	450	0.115	40	1	0.12	0.14	53	49
13	450	0.115	40	0	0.01	0.03	47	51
14	450	0.115	40	1	0.18	0.17	82	87
15	600	0.15	30	0.5	0.13	0.16	52	55
16	450	0.115	40	1	0.11	0.14	81	84
17	450	0.045	40	1	0.14	0.17	64	65
18	450	0.115	40	2	0.17	0.15	60	66
19	600	0.08	50	1.5	0.09	0.11	73	74
20	300	0.15	50	0.5	0.02	0.08	58	59
21	450	0.115	40	1	0.16	0.11	65	69
22	600	0.15	50	1.5	0.17	0.12	10	16
23	750	0.115	40	1	0.05	0.03	76	79
24	450	0.115	60	1	0.13	0.1	60	63
25	150	0.115	40	1	0.06	0.05	87	91
26	600	0.08	30	1.5	0.12	0.1	77	72
27	450	0.115	40	1	0.15	0.11	70	75
28	450	0.185	40	1	0.08	0.11	55	59
29	450	0.115	20	1	0.1	0.08	60	68
30	600	0.15	50	0.5	0.04	0.07	70	76
31	300	0.08	50	0.5	0.11	0.12	72	70

(b) Exp. run no.	Experimental variables				Specific growth rate (h^−1^)		Biodegradability (% biodegradation)	
	2,6D-NT (X_1_)	MgSO_4_·7H_2_O (X_2_)	Temperature (X_3_)	Inoculum size (X_4_)	Exp.	Pred.	Exp.	Pred.
1	500	0.08	30	0.5	0.14	0.16	79	81
2	200	0.08	50	1.5	0.08	0.1	60	63
3	350	0.115	40	1	0.16	0.18	57	55
4	200	0.08	50	0.5	0.12	0.14	80	78
5	350	0.115	40	1	0.15	0.17	55	52
6	200	0.08	30	1.5	0.1	0.12	42	45
7	500	0.15	50	1.5	0.16	0.17	49	53
8	350	0.115	40	2	0.15	0.16	71	75
9	500	0.08	30	1.5	0.13	0.12	82	81
10	650	0.115	40	1	0.07	0.09	61	60
11	350	0.185	40	1	0.05	0.08	57	55
12	200	0.15	30	0.5	0.01	0.04	60	59
13	500	0.15	30	1.5	0.12	0.15	75	78
14	350	0.115	40	1	0.13	0.14	77	80
15	200	0.15	50	1.5	0.14	0.15	22	31
16	500	0.08	50	1.5	0.1	0.13	34	44
17	350	0.115	40	0	0	0.02	13	18
18	350	0.115	40	1	0.15	0.14	78	82
19	500	0.15	30	0.5	0.12	0.15	69	75
20	200	0.15	50	0.5	0.1	0.08	50	54
21	200	0.15	30	1.5	0.08	0.12	66	58
22	350	0.115	40	1	0.17	0.18	71	76
23	200	0.08	30	0.5	0.06	0.08	78	73
24	350	0.115	60	1	0.12	0.11	63	71
25	350	0.115	40	1	0.14	0.12	84	89
26	50	0.115	40	1	0.04	0.07	73	71
27	350	0.115	20	1	0.11	0.13	81	85
28	500	0.15	50	0.5	0.03	0.05	62	68
29	500	0.08	50	0.5	0.06	0.08	76	77
30	350	0.115	40	1	0.15	0.17	78	79
31	350	0.045	40	1	0.1	0.11	84	80

All experiments were carried out in duplicate, and the averages of residual 2,4-DNT/2,6-DNT concentration and culture growth were estimated. The variables whose confidence levels were higher than 95 % (*P* < 0.05) were considered to significantly influence biodegradation and were selected for further optimization by RSM.

### Optimization of identified variables by CCD

To describe the nature of the response surface in the experimental region, a central composite design (Montgomery 1991; Sahoo et al. 2010) was applied. As presented in Supplementary Table 2, factors of highest confidence levels elucidated through PBD experimental design were 2,4-DNT/2,6-DNT (X_1_), MgSO_4_·7H_2_O (X_2_), temperature (X_3_) and inoculum size (X_4_). The total numbers of experimental runs carried out were 2^*k*^ + 2 *k* + *n*_0_, where ‘*k*’ is the number of independent variables and *n*_0_ the number of replicate runs performed at center point of the variables. Thus, a total number of 31 experiments were carried out. The coded values of independent variables were found from equation (Sahoo et al. 2011):

1 $$ X_{\text{i}} = \, \frac{{\Delta U_{\text{i}} - U_{0} }}{\Delta U}\;, $$

where *X*_i_ is the coded level (−*α*, −1, 0, +1 and +*α*) of any independent variable, *U*_i_ is the uncoded/actual level of the independent variable, *U*_0_ is the uncoded level of the independent variable at its center point, and *U* is the step change. In the present case, as per the design, the default α value was taken to be 2.

For fitting the experimental results by response surface regression procedure, the following second-order polynomial equation was used:

2 $$ {\text{Y }} = \beta_{0} + \beta_{ 1} {\text{X}}_{ 1} + \beta_{ 2} {\text{X}}_{ 2} + \beta_{ 3} {\text{X}}_{ 3} + \beta_{ 4} {\text{X}}_{ 4} + \beta_{ 5} {\text{X}}_{ 1} {\text{X}}_{ 2} + \beta_{ 6} {\text{X}}_{ 1} {\text{X}}_{ 3} + \beta_{ 7} {\text{X}}_{ 1} {\text{X}}_{ 4} + \beta_{ 8} {\text{X}}_{ 2} {\text{X}}_{ 3} + \beta_{ 9} {\text{X}}_{ 2} {\text{X}}_{ 4} + \beta_{ 10} {\text{X}}_{ 3} {\text{X}}_{ 4} + \beta_{ 1 1} {\text{X}}_{ 1}^{ 2} + \beta_{ 1 2} {\text{X}}_{ 2}^{ 2} + \beta_{ 1 3} {\text{X}}_{ 3}^{ 2} + \beta_{ 1 4} {\text{X}}_{ 4}^{ 2} $$

where *Y* is the measured or fitted or predicted response (% degradation and the specific growth rate, two responses in this case); X_1_, X_2_, X_3_, X_4_ are the coded independent input variables; *β*_0_ is the intercept term; *β*_1_, *β*_2_, *β*_3_, *β*_4_ are the linear coefficients showing the linear effects; *β*_5_, *β*_6_, *β*_7_, *β*_8_, *β*_9_, *β*_10_ are the cross-product coefficients showing the interaction effects; and *β*_11_, *β*_12_, *β*_13_, *β*_14_ are the quadratic coefficients showing the squared effects cross-product coefficients showing the interaction effects.

### Experimental validation of the statistical model

To check the validation of model predictions, a set of shake-flask experiments at optimal factor levels were run, and the experimental and predicted % biodegradation and specific growth rate were compared. The validation run experiments were conducted in 250-ml conical flasks containing 100 ml aliquots of the optimized medium. The samples were withdrawn aseptically at appropriate intervals and further analyzed for biomass (O.D.), dry cell weight and residual concentration of DNTs.

### Analytical methods

Biomass in the samples was determined by measuring its optical density (O.D.) at 600 nm using a UV–visible spectrophotometer. The O.D. value was then converted to dry cell mass using appropriate calibration curve by plotting dry weight of biomass per liter against optical density of the suspension. To determine the amount of remaining 2,4- and 2,6-DNT, samples were withdrawn at regular intervals from the MSB media, centrifuged (6000*g*, 15 min) (Hereaus, Kendro Laboratory Products, Germany) and extracted twice with diethyl ether. Each sample extract was evaporated to dryness at 30 °C and redissolved in 0.5 ml methanol and was quantitatively analyzed by a HPLC equipped with UV (254 nm) detector using C_18_ column (4.6 × 250 mm; particle size: 5 µm) (Young Lin Autochro, S. Korea) using solvent methanol/water (60:40, v/v) as mobile phase at the flow rate of 1 ml min^−1^ (Mulla et al. 2013). Under these conditions, retention times of 2,4-DNT and 2,6-DNT were 31 and 29 min, respectively. Percentage biodegradation was calculated by applying the following formula:

3 $$ {\text{Residual DNTs (}}\% ) { } = \, \left( {\frac{{C_{t} }}{{C_{0} }}} \right) \times 100\;, $$

where *C*_0_ is the initial concentration of DNTs in the medium, and *C*_*t*_ is the concentration at time *t*.

### Statistical analysis

Experiments were carried out in duplicate, and final data were reported in terms of mean values. Statistical experimental designs were generated and analyzed using the statistical software package, Reliasoft Design of Experiment (DOE++; v 9.0.13) with a risk factor (*α*) of 0.05 (i.e. 95 % level of confidence) for both PBD and CCD. Three-dimensional surface plots were constructed for visualization of interaction between significant variables and their optimal values. Statistical analysis of the model was performed to evaluate the analysis of variance (ANOVA). The quality of the polynomial model equation was judged statistically by the coefficient of determination *R* ^2^, and its statistical significance was determined by an *F* test. The significance of the regression coefficients was tested by a *t* test. Desirability function (Sahoo et al. 2010, 2011) was used for simultaneous optimization of all affecting parameters to achieve the highest % degradation and specific growth rate.

## Results and discussion

In this study, the MSB medium was optimized statistically for optimal degradation of DNTs by *R. pyridinivorans* NT2. According to the results from the literature review and preliminary studies, 2,4-DNT/2,6-DNT (X_1_), MgSO_4_ (X_2_), K_2_HPO_4_ (X_3_), KH_2_PO_4_ (X_4_), yeast extract (X_5_), temperature (X_6_), pH (X_7_), agitation speed (X_8_), and inoculum size, i.e., OD (X_9_) were selected to optimize the medium composition using 12-run PBD. The analysis of PBD leads us to identify the most significant factors to proceed to the second phase of experimentation.

### Screening of media constituents and physical parameters using the two-level factorial design

All the nine cultivation parameters were investigated for their effects on biodegradation and specific growth rate of *R. pyridinivorans* NT2 by taking 95 % as confidence level (*α* = 0.05) in statistical analysis of the results. Supplementary Tables 3 and 4 present the ANOVA and regression analysis of percentage degradation and specific growth rate, respectively. ANOVA for percentage degradation and specific growth rate indicated the *F* values to be 39.32 and 28.63 (2,4-DNT), 22.25 and 41.08 (2,6-DNT), respectively, and implied that the model was significant. This was further supported by a low value of *P* (<0.05). Based on regression analysis of the model, the coefficient of determination (*R* ^2^) values were 99.44 % and 99.23 % (2,4-DNT), 99.01 and 99.46 % (2,6-DNT), along with adjusted *R* ^2^ values of 96.91 and 95.76 % (2,4-DNT), 94.56 and 97.04 % (2,6-DNT) for percentage degradation and specific growth rate, respectively. These values again ensured a satisfactory adjustment of the model to the experimental data. The order of significantly affected variables in 2,4-DNT degradation was: inoculum size (OD) > 2,4-DNT > MgSO_4_·7H_2_O > temperature (specific growth rate) and inoculum size (OD) > MgSO_4_·7H_2_O > temperature > 2,4-DNT (% biodegradation). The pattern of response of specific growth rate and biodegradability (% biodegradation) in 2,6-DNT was similar with the data of 2,4-DNT degradation. All the other factors with *P* value >0.05 did not significantly affect the biodegradation of DNTs or the culture specific growth rate and, thus, were ruled out.

According to ANOVA, regression analysis, mean effect plots (Supplementary Fig. 1) and Pareto charts (Supplementary Fig. 2), the four factors, inoculum size (OD), MgSO_4_·7H_2_O, 2,4-DNT/2,6-DNT and temperature, which had higher significance effect on both  % biodegradation and specific growth rate, were chosen for further optimization of their levels. The remaining factors (agitation speed, pH, concentrations of K_2_HPO_4_, KH_2_PO_4_ and yeast extract) were fixed at the levels where *Y*_% degradation_ and *Y*_specific growth rate_ were maximum. From the mean effect plots, it can be observed that the higher system responses were obtained at the highest level of MgSO_4_·7H_2_O (0.1 g l^−1^), KH_2_PO_4_ (2 g l^−1^), yeast extract (1 g l^−1^), temperature (40 °C), pH (9) and inoculums size (1 %) and the lowest level of K_2_HPO_4_ (0.5 g l^−1^) and agitation speed (110 rpm).

### RSM approach for optimization of operational parameters

The CCD comprises three groups of design points: two-level factorial design points, axial points and center points. Thus, the relationship and interrelationship of factors were determined by fitting the second-order polynomial equation to the data obtained from the CCD experiments. Table 2 presents the matrix of the CCD design with actual values for independent variables and the corresponding two responses (specific growth rate and percentage biodegradation).

For maximizing biodegradability, the two DNT and the culture specific growth rate, the levels of the four screened process variables, i.e., inoculum size (OD), MgSO_4_·7H_2_O, 2,4-DNT/2,6-DNT and temperature, were varied using the CCD. The four independent variables were studied at five different levels (−*α*, −1, 0, +1, +*α*), and a set of 31 experiments were carried out. A multiple regression analysis of the data was carried out to obtain an empirical model that relates the measured response to the independent variables.

Based on the results obtained, regression model equations were developed for depicting the relationship between the various medium constituents and the responses on percentage biodegradation and specific growth rate. The second-order regression equation for 2,4-DNT degradation was:

4 $$ {\text{Y}}_{ 1} f\left( {\text{X}} \right) \, = { 76}. 3 1 7 3 { } + \, 0. 2 6 2 5 {\text{X}}_{ 1} - 1 6 1. 4 1 8 9 {\text{X}}_{ 2} - 2. 6 9 5 8 {\text{X}}_{ 3} + { 8}. 9 8 8 1 {\text{X}}_{ 4} - 0. 7 7 3 8 {\text{X}}_{ 1} {\text{X}}_{ 2} + \, 0.00 3 5 {\text{X}}_{ 1} {\text{X}}_{ 3} - 0.0 8 2 5 {\text{X}}_{ 1} {\text{X}}_{ 4} + { 24}. 4 6 4 3 {\text{X}}_{ 2} {\text{X}}_{ 3} - 2 7 5.0000{\text{X}}_{ 2} {\text{X}}_{ 4} + { 2}.0 8 7 5 {\text{X}}_{ 3} {\text{X}}_{ 4} - 0.000 2 {\text{X}}_{ 1}^{ 2} - 900. 1 4 5 8 {\text{X}}_{ 2}^{ 2} - 0.0 4 4 8 {\text{X}}_{ 3}^{ 2} - 1 5. 4 10 7 {\text{X}}_{ 4}^{ 2} $$

5 $$ {\text{Y}}_{2} f(X) \, = - 0.3977 \, + \, 0.0014{\text{X}}_{1} + \, 0.5279{\text{X}}_{2} + \, 0.0083{\text{X}}_{3} + \, 0.0483{\text{X}}_{4} - 0.0001{\text{X}}_{1} {\text{X}}_{2} - 4.5833{\text{E}} - 06{\text{X}}_{1} {\text{X}}_{3} - 0.0003{\text{X}}_{1} {\text{X}}_{4} - 0.0268{\text{X}}_{2} {\text{X}}_{3} + \, 1.5357{\text{X}}_{2} {\text{X}}_{4} + \, 0.0016{\text{X}}_{3} {\text{X}}_{4} - 9.0939{\text{E}} - 07{\text{X}}_{1}^{2} - 5.4786{\text{X}}_{2}^{2} - 5.4613{\text{E}} - 05{\text{X}}_{3}^{2} - 0.0468{\text{X}}_{4}^{2} $$

The fitted second-order response surface models specified by Eq. (2) for degradation of 2,6-DNT in terms of actual process variables was:

6 $$ {\text{Y}}_{ 1} f({\text{X}}) \, = { 87}. 3 9 4 4 { } + \, 0. 2 2 4 2 {\text{X}}_{ 1} - 2 9 3. 5 8 60{\text{X}}_{ 2} - 1. 7 6 3 1 {\text{X}}_{ 3} + { 3}. 7 3 8 1 {\text{X}}_{ 4} - 0. 5 7 1 4 {\text{X}}_{ 1} {\text{X}}_{ 2} + \, 0.00 2 8 {\text{X}}_{ 1} {\text{X}}_{ 3} - 0.0 6 8 3 {\text{X}}_{ 1} {\text{X}}_{ 4} + { 21}.0 7 1 4 {\text{X}}_{ 2} {\text{X}}_{ 3} - 1 9 2. 8 5 7 1 {\text{X}}_{ 2} {\text{X}}_{ 4} + { 1}. 8 500{\text{X}}_{ 3} {\text{X}}_{ 4} - 0.000 3 {\text{X}}_{ 1}^{ 2} - 7 6 2. 8 7 6 6 {\text{X}}_{ 2}^{ 2} - 0.0 4 1 8 {\text{X}}_{ 3}^{ 2} - 1 8. 7 3 8 1 {\text{X}}_{ 4}^{ 2} $$

7 $$ {\text{Y}}_{2} f\left( {\text{X}} \right) \, = - 0.2695 \, + \, 0.0012{\text{X}}_{1} + \, 0.7483{\text{X}}_{2} + \, 0.0057{\text{X}}_{3} + \, 0.0392{\text{X}}_{4} + \, 0.0004{\text{X}}_{1} {\text{X}}_{2} - 1.4583{\text{E}} - 05{\text{X}}_{1} {\text{X}}_{3} + \, 4.1667{\text{E}} - 05{\text{X}}_{1} {\text{X}}_{4} + \, 0.0304{\text{X}}_{2} {\text{X}}_{3} + \, 0.7500{\text{X}}_{2} {\text{X}}_{4} + \, 0.0009{\text{X}}_{3} {\text{X}}_{4} - 9.3056{\text{E}} - 07{\text{X}}_{1}^{2} - 13.0102{\text{X}}_{2}^{2} - 5.9375{\text{E}} - 05{\text{X}}_{3}^{2} - 0.0637{\text{X}}_{4}^{2} $$

where Y_1_ = percentage biodegradation, Y_2_ = specific growth rate, X_1_ is 2,4-DNT/2,6-DNT, X_2_ is MgSO_4_·7H_2_O, X_3_ is temperature and X_4_ is inoculum size (OD). The negative and positive signs of regression coefficients indicate the antagonistic effect and synergistic effect of each variable, respectively.

The statistical significance of Eqs. (4–7) was checked by *F* test. The ANOVA and student’s *t* test of the models for percentage biodegradation and specific growth rate were obtained as before in the screening study, and are given in Tables 3 and 4, respectively. In 2,4-DNT biodegradation, squared model terms X_1_^2^, X_3_^2^ and interaction of X_1_ with X_4,_ X_2_ with X_3_, X_3_ with X_4_ were highly significant (*P* < 0.001) while calculating biodegradability (% biodegradation). In terms of specific growth rate, coefficient of linear term X_4_, squared model term X_1_^2^ and interaction of X_1_ with X_4_ as well as X_2_ with X_4_ were highly significant (*P* < 0.001). Similarly, squared model terms X_1_^2^, X_3_^2^ and X_4_^2^, and interaction of X_1_ with X_4_, X_2_ with X_3_, X_3_ with X_4_ were highly significant (*P* < 0.001) for biodegradability (% biodegradation) of 2,6-DNT. However, the coefficient of X_4_, squared model term X_1_^2^, X_2_^2^ and X_4_^2^ and the two-way interaction between X_1_ and X_3_ were significant model terms in the calculation of specific growth rate of 2,6-DNT biodegradation studies.

**Table 3:** ANOVA of percentage degradation and specific growth rate in the optimization study of (a) 2,4-DNT and (b) 2,6-DNT biodegradation

	Specific growth rate (h^−1^)					Biodegradability (% biodegradation)				
	*df*	SS	MS	*F*	*P* (Prob >*F*)	*df*	SS	MS	*F*	*P* (Prob >*F*)
(a) Source of variation										
Model	14	0.0622	0.0044	4.4744	0.0027	14	6575.9236	469.7088	4.0448	0.0046
Main effects	4	0.0237	0.0059	5.975	0.0039	4	440.1667	110.0417	0.9476	0.4622
Two-way interactions	6	0.0233	0.0039	3.9196	0.0134	6	4593.875	765.6458	6.5932	0.0012
Quadratic effects	4	0.0151	0.0038	3.806	0.0233	4	1541.8819	385.4705	3.3194	0.0368
Residual	16	0.0159	0.001			16	1858.0119	116.1257		
Lack of fit	10	0.0114	0.0011	1.5237	0.3137	10	1644.5833	164.4583	4.6233	0.0371
Pure error	6	0.0045	0.0007			6	213.4286	35.5714		
Total	30	0.078				30	8433.9355			
* R* ^2^ 0.7965, Adj *R* ^2^ 0.6185						*R* ^2^ 0.7797, Adj *R* ^2^ 0.5869				
(b) Source of variation										
Model	14	0.0518	0.0037	4.9429	0.0016	14	5419.3072	387.0934	3.9701	0.005
Main effects	4	0.0165	0.0041	5.5282	0.0054	4	383	95.75	0.982	0.445
Two-way interactions	6	0.0127	0.0021	2.8365	0.0446	6	3258	543	5.5691	0.0028
Quadratic effects	4	0.0225	0.0056	7.5174	0.0013	4	1778.3072	444.5768	4.5596	0.012
Residual	16	0.012	0.0007			16	1560.0476	97.503		
Lack of fit	10	0.011	0.0011	6.585	0.0158	10	1442.3333	144.2333	7.3517	0.0119
Pure error	6	0.001	0.0002			6	117.7143	19.619		
Total	30	0.0638				30	6979.3548			
* R* ^2^ 0.8122, Adj *R* ^2^ 0.6479						*R* ^2^ 0.7765, Adj *R* ^2^ 0.5809				

*df* degrees of freedom, SS sum of squares, MS mean sum of squares

**Table 4:** Estimated regression coefficients and their significance in the models in the optimization study of (a) 2,4-DNT and (b) 2,6-DNT biodegradation

	Specific growth rate (h^−1^)				Biodegradability (% biodegradation)			
	Coefficient	Standard error	*T* value	*P* value	Coefficient	Standard error	*T* value	*P* value
(a) Term								
Intercept	0.1719	0.0153	17.7267	<0.0001	95.8743	3.6433	27.3686	<0.0001
X_1_:2,4-DNT	0.0044	0.0059	0.7624	0.3066	1.5711	1.6622	0.8879	0.4051
X_2_:MgSO_4_	−0.0023	0.0059	−0.4462	0.6298	−2.388	1.6622	−1.3105	0.2267
X_3_:Temp	−0.0011	0.0059	−0.2155	0.7311	−0.127	1.6622	−0.0784	0.9175
X_4_:Inoculum	0.0258	0.0059	4.3711	<0.0001	0.2955	1.6622	0.1666	0.8755
X_1_X_2_	−0.0043	0.0077	−0.6102	0.5709	−0.8465	2.208	−0.3343	0.7477
X_1_X_3_	−0.0152	0.0077	−2.8022	0.3104	−1.9328	2.208	−0.8548	0.393
X_1_X_4_	0.0087	0.0077	1.1442	<0.0001	0.3119	2.208	0.1213	<0.0001
X_2_X_3_	0.0063	0.0077	0.9573	0.3018	6.6155	2.208	3.0827	<0.0001
X_2_X_4_	0.0116	0.0077	1.6525	<0.0001	3.5625	2.208	1.6263	0.1265
X_3_X_4_	0.0063	0.0077	0.9883	0.3522	0.4772	2.208	0.1541	<0.0001
X_1_^2^	−0.0233	0.0056	−4.4962	<0.0001	−16.5628	1.6333	−10.4247	<0.0001
X_2_^2^	−0.0184	0.0056	−3.6041	0.0589	−8.4475	1.6333	−5.0057	0.0457
X_3_^2^	−0.0084	0.0056	−1.6293	0.1106	−3.1755	1.6333	−1.8346	<0.0001
X_4_^2^	−0.0184	0.0056	−3.6053	<0.0001	−2.4622	1.6333	−1.5866	0.1403
(b) Term								
Intercept	0.2114	0.0178	16.7369	<0.0001	94.8663	3.5999	28.3772	<0.0001
X_1_:2,6-DNT	0.0041	0.0053	0.7829	0.3018	1.5414	1.7587	0.8374	0.4075
X_2_:MgSO_4_	−0.0028	0.0053	−0.4982	0.6251	−2.335	1.7437	−1.3664	0.2022
X_3_:Temp	−0.0014	0.0061	−0.2135	0.8336	−0.125	1.6922	−0.0795	0.9351
X_4_:Inoculum	0.0259	0.0058	4.3415	<0.0001	0.2715	1.6922	0.1172	0.8732
X_1_X_2_	−0.0044	0.0072	−0.6102	0.5503	−0.8235	2.205	−0.3933	0.7176
X_1_X_3_	−0.0193	0.0072	−2.7022	<0.0001	−1.9388	2.205	−0.8525	0.344
X_1_X_4_	0.0088	0.0072	1.1332	0.5168	0.3223	2.205	0.1434	<0.0001
X_2_X_3_	0.0069	0.0072	0.9589	0.3519	6.8455	2.205	3.0924	<0.0001
X_2_X_4_	0.0121	0.0072	1.6562	<0.0001	3.5576	2.205	1.6176	0.1297
X_3_X_4_	0.0067	0.0072	0.9589	0.3519	0.4672	2.205	0.1887	<0.0001
X_1_^2^	−0.0241	0.0054	−4.4366	<0.0001	−18.3124	1.6874	−10.2516	<0.0001
X_2_^2^	−0.0178	0.0054	−3.5043	<0.0001	−8.4729	1.6874	−5.0484	0.0441
X_3_^2^	−0.0078	0.0054	−1.6398	0.1206	−3.1921	1.6874	−1.8221	<0.0001
X_4_^2^	−0.0178	0.0054	−3.5043	<0.0001	−2.2524	1.6874	−1.5188	<0.0001

The coefficient of determination (*R* ^2^) value ranged between 77 and 90 % for both percentage biodegradation and specific growth rate in case of both DNTs. The values of *R* ^2^ and adjusted *R* ^2^ were close to 1.0 and advocated a high correlation between the observed values and the predicted values. Adequate precision is a measure of the signal-to-noise ratio, and a value greater than 4.0 is desirable. The adequate precision value ranged between 8.81 and 10.77, which indicated an adequate signal and suggested that the model can be used to navigate the design space. Thus, it can be concluded that there is a good agreement between the experimental values and the second-order polynomial model developed, and the observed differences (i.e., the residuals) may be readily explained as random noise (Tsimas et al. 2009; Tzikalos et al. 2013).

**Fig. 1:**
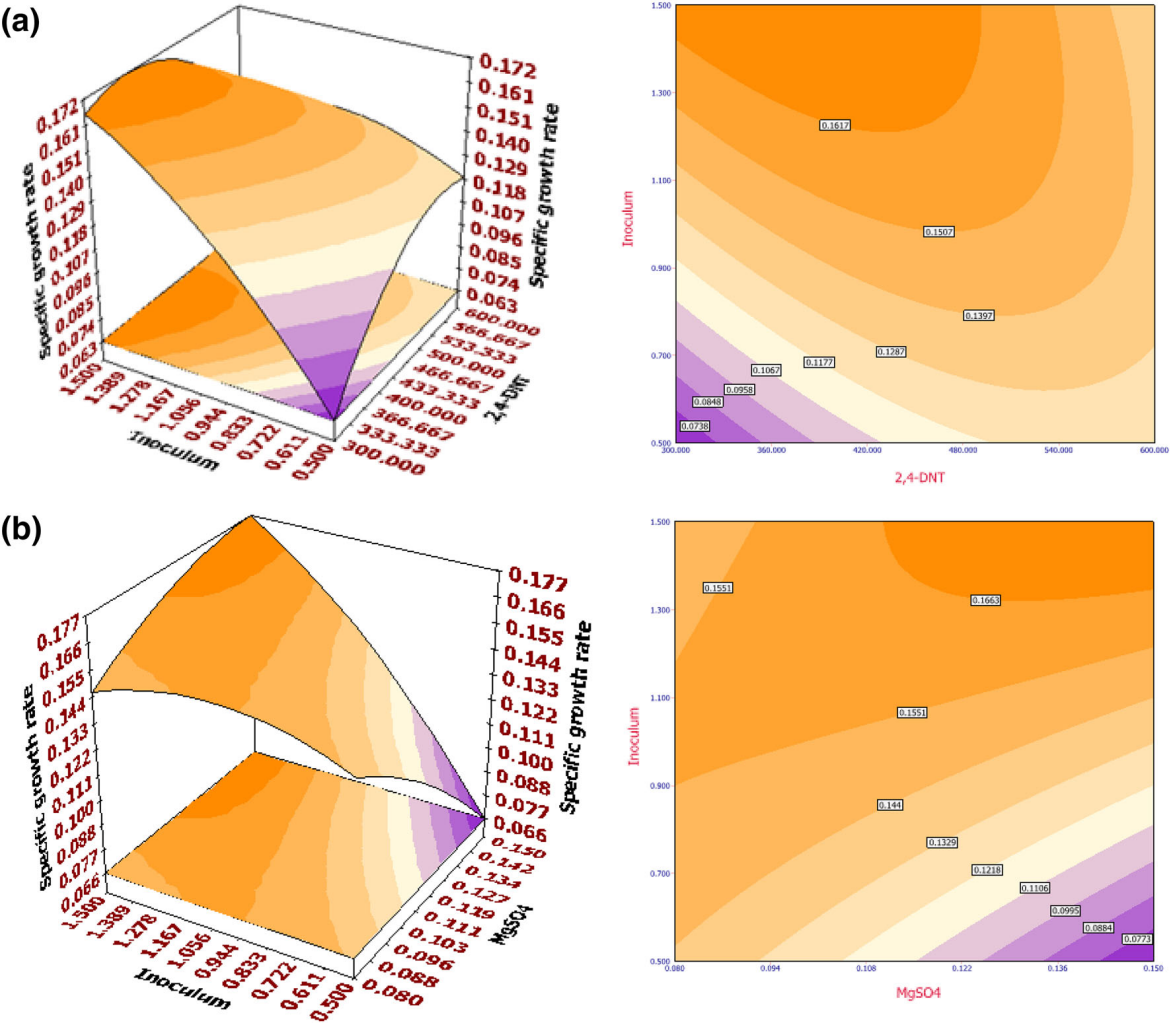
3D surface (*left*) and 2D contour (*right*) plot for the response of specific growth rate of *R. pyridinivorans* NT2 in 2,4-DNT biodegradation as a function of **a** inoculum size (OD) and initial 2,4-DNT concentration (mg l^−1^) and **b** inoculum size (OD) and MgSO_4_·7H_2_O (g l^−1^)

**Fig. 2:**
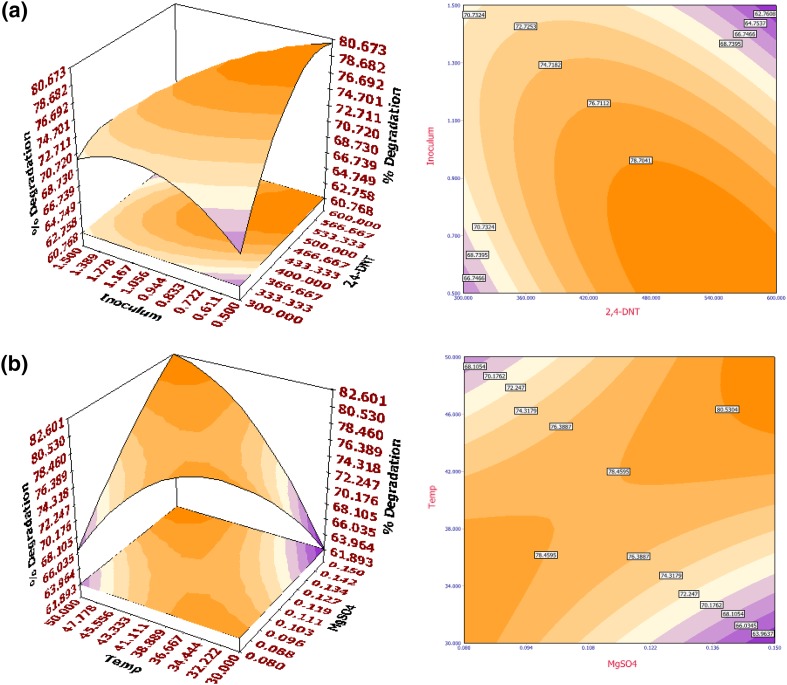
3D surface (*left*) and 2D contour (*right*) plot showing the behavior of  % biodegradation as a function of **a** inoculum size (OD) and initial 2,4-DNT concentration (mg l^−1^) and **b** MgSO_4_·7H_2_O (g l^−1^) and temperature (°C) during 2,4-DNT biodegradation

**Fig. 3:**
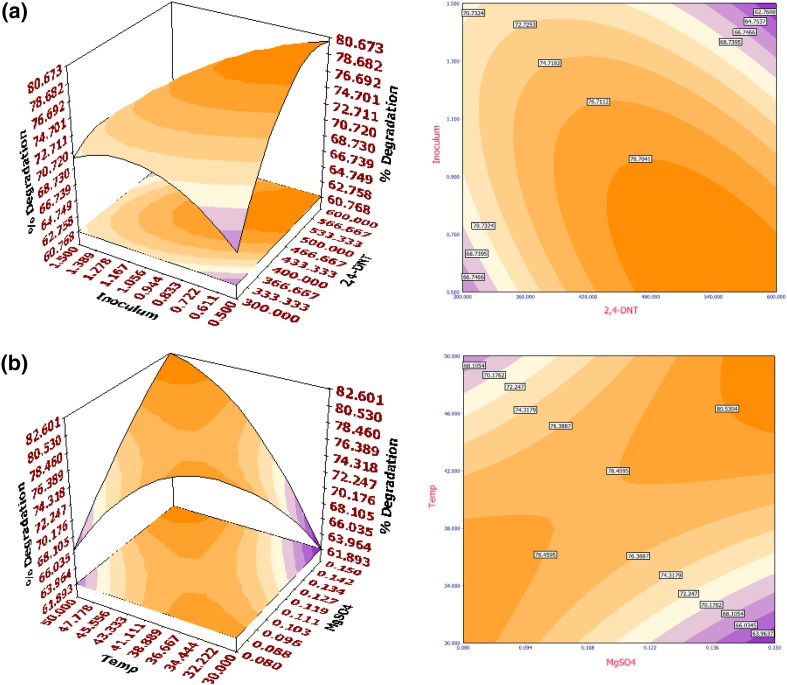
3D surface (*left*) and 2D contour (*right*) plot for the response of specific growth rate of *R. pyridinivorans* NT2 in 2,6-DNT biodegradation as a function of **a** initial 2,6-DNT concentration (mg l^−1^) and temperature (°C) and **b** MgSO_4_·7H_2_O (g l^−1^) and temperature (°C)

The fitted response surface (3D) and their corresponding contour (2D) plots for percentage biodegradation and specific growth rate by the aforementioned model were generated to investigate the individual and cumulative effects of 2,4-DNT/2,6-DNT, MgSO_4_·7H_2_O, temperature and inoculum size (OD). The surface plots are generated for the pair-wise combination of factors with significant mutual effects, while other factors are set at their middle (0) levels. From the nature of the response surface contours, whether elliptical, circular or saddle point, interaction between the variables may be predicted. Interaction of inoculum size (OD) with 2,4-DNT concentration (Fig. 1a) and MgSO_4_·7H_2_O (Fig. 1b) (on specific growth rate) and interaction of 2,4-DNT concentration with inoculum size (OD) (Fig. 2a), MgSO_4_·7H_2_O with temperature (Fig. 2b) as well as temperature with inoculum size (OD) (Supplementary Fig. 3) had significant impact among variables on  % biodegradation. For specific growth rate in 2,6-DNT degradation, mutual interactions between the variables concentration of 2,6-DNT with temperature (Fig. 3a) and MgSO_4_·7H_2_O with temperature (Fig. 3b) were the most significant. On the other hand, interactions of inoculum size (OD) with 2,6-DNT concentration (Supplementary Fig. 4), and MgSO_4_·7H_2_O with temperature (Supplementary Fig. 5) were of highest significance on % biodegradation of 2,6-DNT.

To determine the optimal levels of each variable for maximizing both % biodegradation and the culture specific growth rate, the method of desirability function was applied. The desirability function test in this multiple response optimization reveals that the overall desirability function for biodegradation of DNTs and specific growth rate are close to 1, indicating that the function increases linearly toward the desired target values of the two responses. Besides, individual desirability values of the two responses were calculated and were found to be 1 each for the two responses. Using the desirability function method for optimizing both the responses simultaneously, optimum values of the cultivation conditions were estimated as follows: 2,4-DNT/2,6-DNT = 474/470 mg l^−1^, MgSO_4_·7H_2_O = 0.11 g l^−1^, temperature = 37.5 °C, and inoculum size = 1.05 OD, all of which were located within the experimental range. The predicted responses as maximum % degradation and specific growth rate under these optimum conditions were 97.55 % and 0.19 h^−1^, respectively.

### Validation of the experimental model under optimized settings

**Fig. 4:**
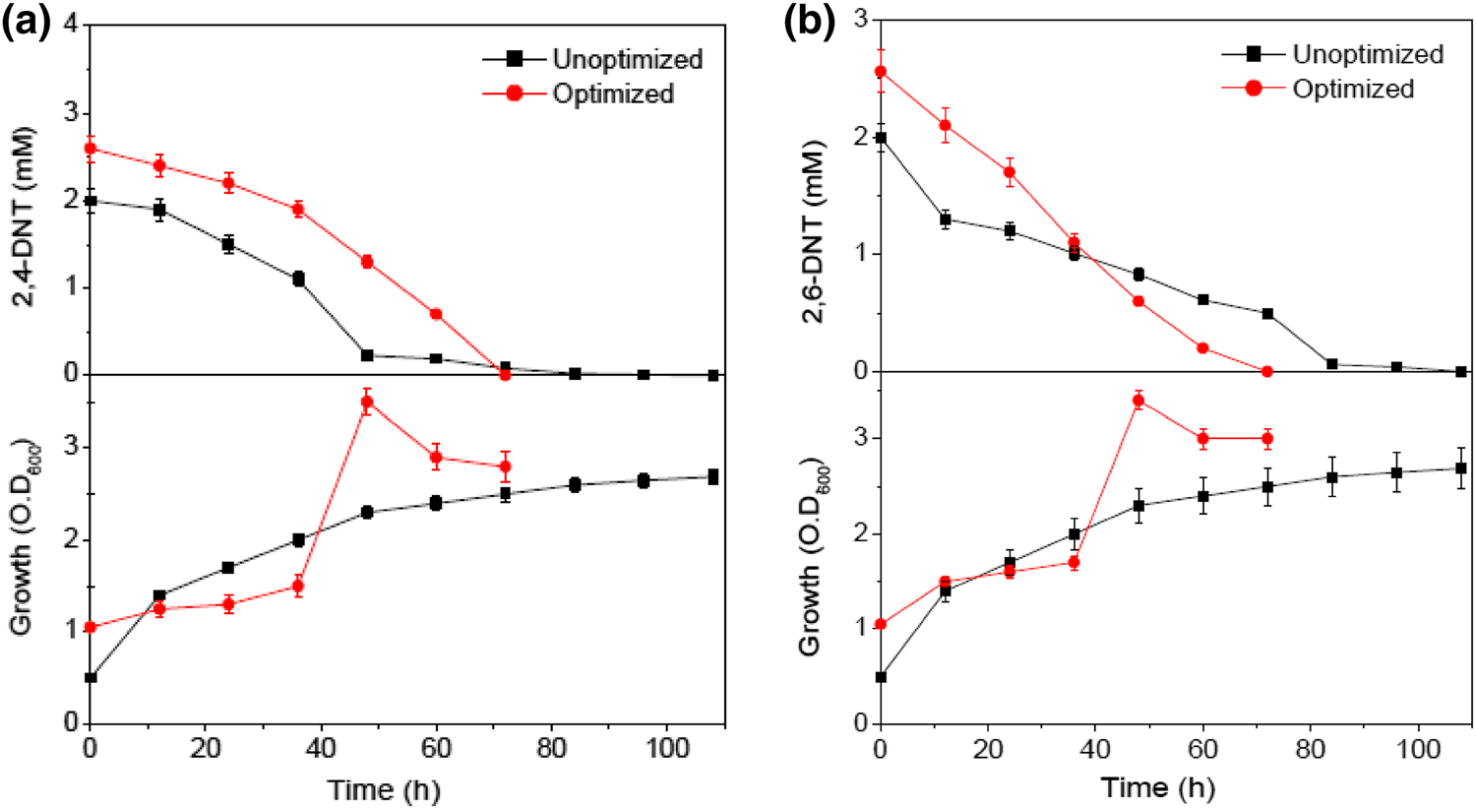
Validation experiments of **a** 2,4-DNT and **b** 2,6-DNT biodegradation by *R. pyridinivorans* NT2 in optimal (*red filled circle*) and previous (*black filled square*) MSB media. Data are mean values of two batches. *Vertical bars* are standard errors; where absent, *bars* fall within *symbols*

To confirm the validity and accuracy of the model, additional confirmation experiments were done in triplicates according to the optimum configuration in batch shake-flask. 2,4- and 2,6-DNT were degraded at 0.036 and 0.035 mM h^−1^ with a specific growth rate of 0.166 and 0.141 h^−1^, respectively (Fig. 4). Using the previous unoptimized MSB media, the strain NT2 could degrade 365 mg l^−1^ (2 mM) and 275 mg l^−1^ (1.5 mM) of 2,4-DNT and 2,6-DNT, respectively. Thus, these optimized settings resulted in (1) 30 % (2,4-DNT) and 70 % (2,6-DNT) increased biodegradation, (2) 5.64-fold increase in specific growth rate for both DNTs, and (3) significant reduction in total degradation time (108 h in unoptimized vs. 72 h in optimized). The comparable predicted and experimental system responses reflect the accuracy and applicability of RSM for optimizing the biodegradation process.

The biodegradability (% biodegradation) of 2,4-DNT and 2,6-DNT obtained here was compared with previous published data. Supplementary Table 5 shows the biodegradation of DNTs under aerobic and anaerobic conditions. Using the optimization medium, 100 % of both 2,4-DNT and 2,6-DNT was degraded in 72 h at 0.036 and 0.035 mM h^−1^, respectively. In other studies, 2,4-DNT and 2,6-DNT were degraded within a range of 0.0001–0.0604 mM h^−1^. Hughes et al. (1999) reported a higher value; however, *Clostridium acetobutylicum* was used to grow in anaerobic condition, compared to aerobic growth in this study. Moreover, despite DNT’s ability to serve as a nitrogen source and even as an energy source, the biotransformation of DNTs requires a primary substrate to serve as a carbon source and reducing equivalents in most cases (Wittich et al. 2009; Chien et al. 2014). Further, this is the first report of optimization of DNT degradation where the biodegradation time was reduced by 36 h, which should be cost-effective on a large-scale operation. Also, the decontaminated extracts may be used as a soil conditioner due to the content of organic carbon, total nitrogen, potassium as K_2_O and phosphorus as P_2_O_5_ (Yuan et al. 2006).

## Conclusions

This study is the first report employing central composite design and response surface methodology to optimize the nutritional and physical parameters for enhanced biodegradation of DNTs. The results clearly demonstrated enhancement in biodegradability (% biodegradation) of 2,4-DNT and 2,6-DNT by *R. pyridinivorans* isolate NT2 following screening and optimization of medium constituents. Based on the Plackett–Burman design for screening the medium constituents, the substrates tested (2,4-DNT, 2,6-DNT), MgSO_4_·7H_2_O, temperature and inoculum size (OD) were found to be the most influential factors affecting biodegradability (% biodegradation) and specific growth rate. At the RSM optimized levels, increased biodegradation (2,4-DNT: 30 % and 2,6-DNT: 70 %) and increased specific growth rate (5.64-fold) were achieved in 33 % less time. Further pilot scale studies are required with this strain for actual applications in industrial wastewaters, and it is set aside for the next study.

## Electronic supplementary material

Below is the link to the electronic supplementary material.

Supplementary material 1 (DOCX 918 kb)
