# Supplementary material for: Statistical modeling and optimization of culture conditions by response surface methodology for 2,4- and 2,6-dinitrotoluene biodegradation using Rhodococcus pyridinivorans NT2
Source: 3 Biotech. 2016 Jul 19;6(2):155. doi: 10.1007/s13205-016-0468-9 (PMC4951380; doi:10.1007/s13205-016-0468-9)
Supplement: Supplementary file 1 — Supplementary material 1 (DOCX 918 kb) [file 13205_2016_468_MOESM1_ESM.docx]

**Supplementary data**

Statistical modelling and optimization of culture conditions by response surface methodology for 2,4- and 2,6-dinitrotoluene biodegradation using *Rhodococcus pyridinivorans* NT2

Debasree Kundu, Chinmay Hazra and Ambalal Chaudhari

School of Life Sciences, North Maharashtra University, Jalgaon 425 001, Maharashtra, India

* Corresponding author. E-mail: ambchasls@gmail.com; ambchasls@yahoo.com Tel.: +91 257 2257425

**Fig. 1 (a):** Mean effect plots of the 9 factors for specific growth rate in the screening study of degradation of (a) 2,4-DNT and (b) 2,6-DNT.


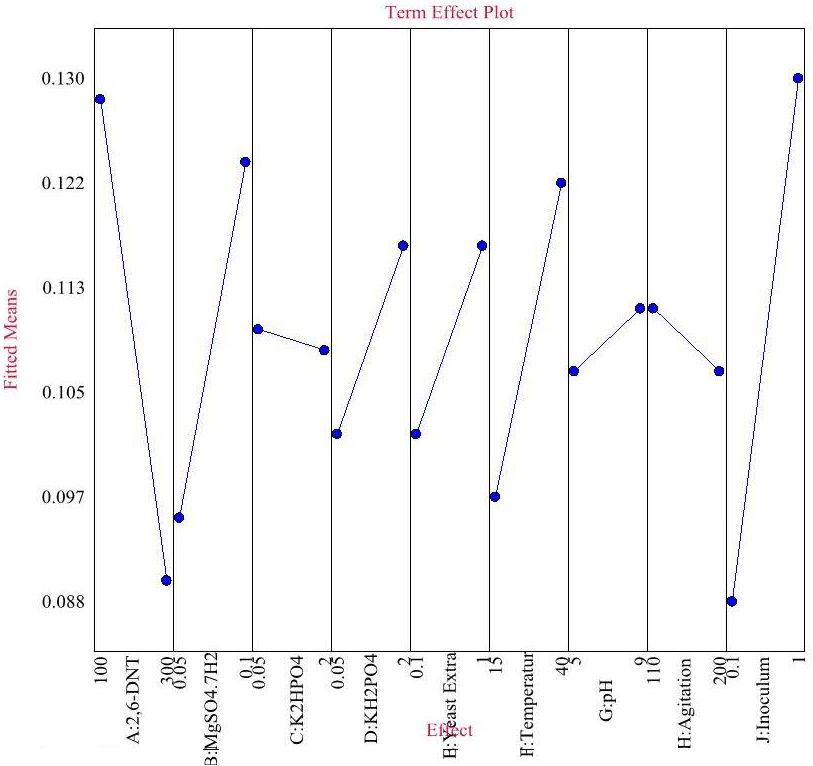

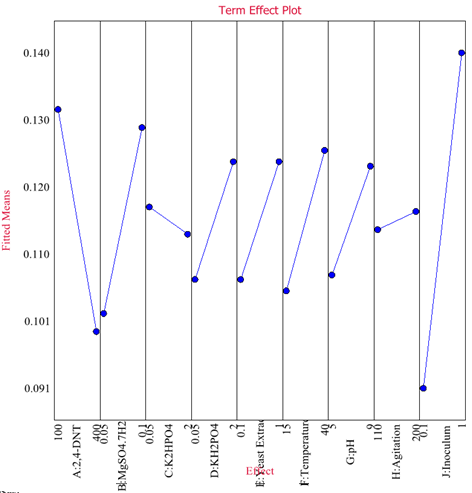


(b)

(a)

**
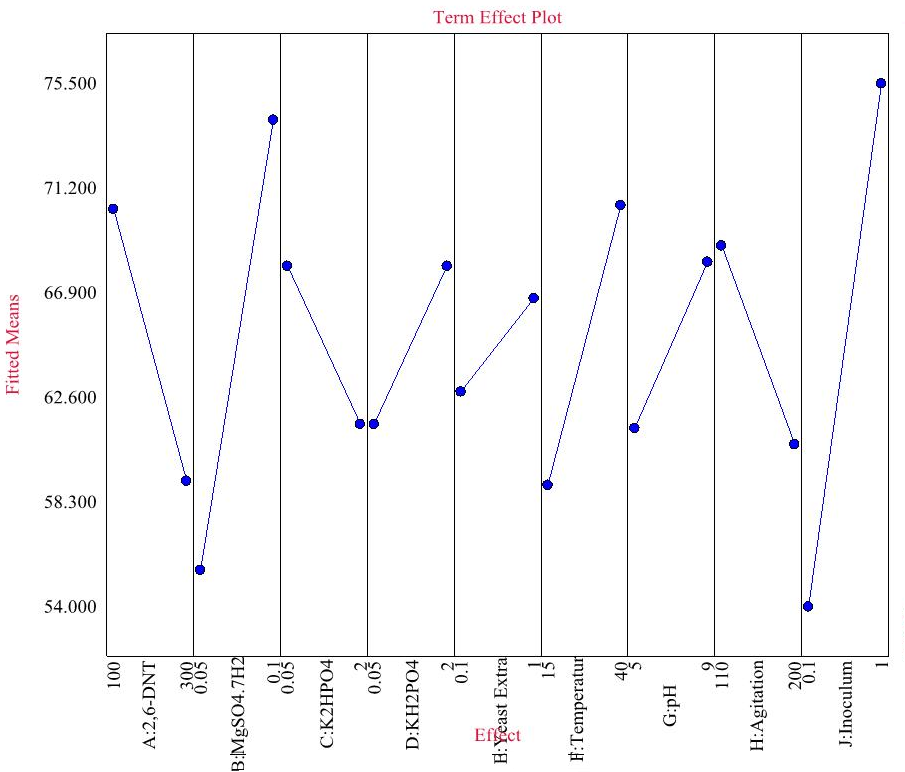

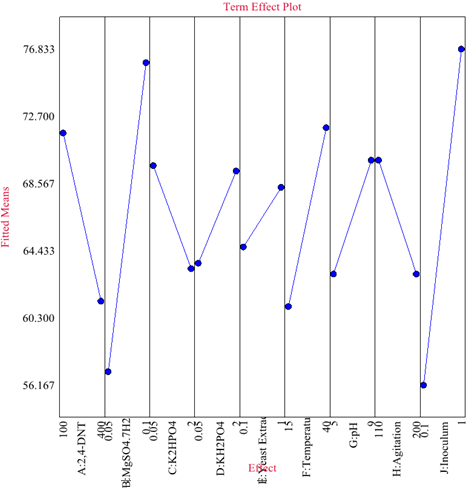
Fig. 1 (b):** Mean effect plots of the 9 factors for biodegradation rate in the screening study of degradation of (a) 2,4-DNT and (b) 2,6-DNT.

(a)

(b)

**Fig. 2 (a):** Pareto chart of Placket-Burman design for specific growth rate during degradation of (a) 2,4-DNT and (b) 2,6-DNT.


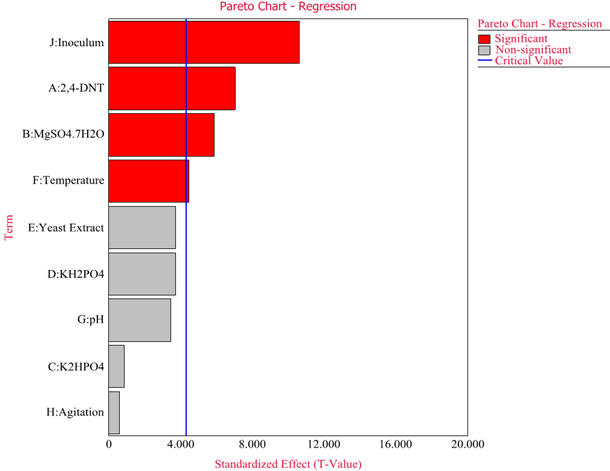

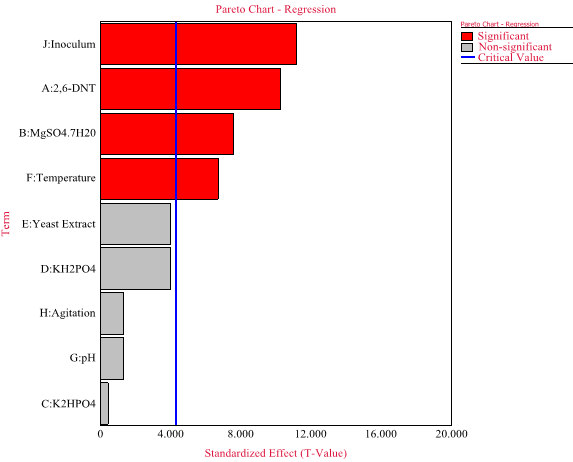


(b)

(a)

**Fig. 2 (b):** Pareto chart of Placket-Burman design for degradation rate of (a) 2,4-DNT and (b) 2,6-DNT

(a)

(b)


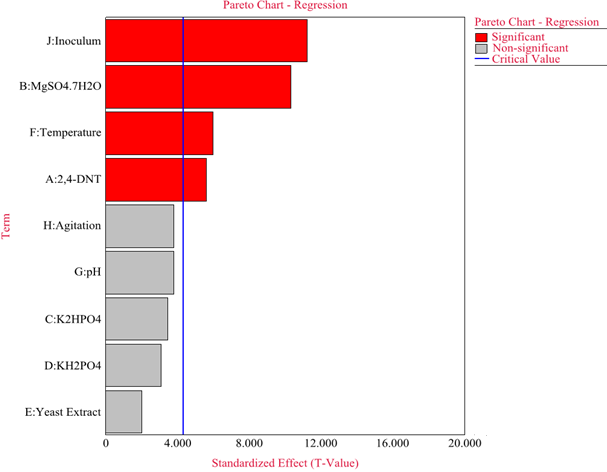

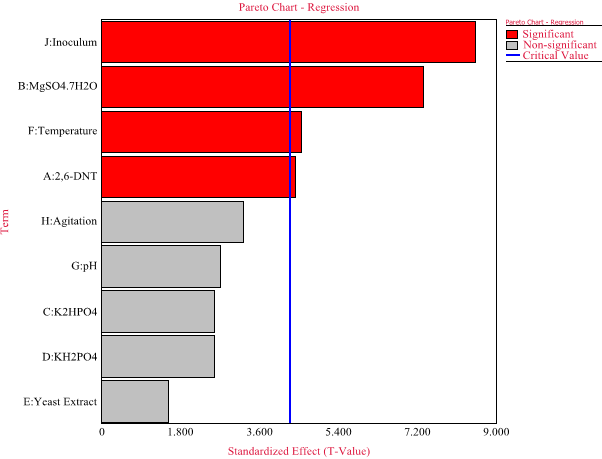


**Fig. 3:** 3D surface (left) and 2D contour (right) plot for the response of % biodegradation of 2,4-DNT by *R. pyridinivorans* NT2 as a function of inoculum size (OD) and temperature.


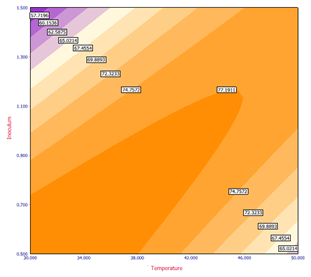

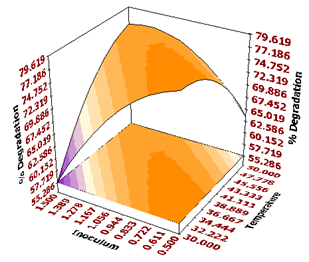


**Fig. 4:** 3D surface (left) and 2D contour (right) plot showing the behavior of % biodegradation as a function of inoculum size (OD) and initial 2,6-DNT concentration.

**
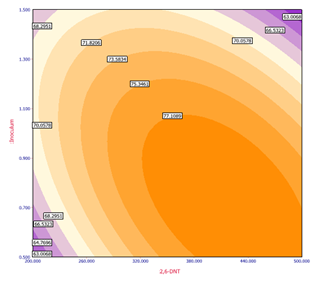

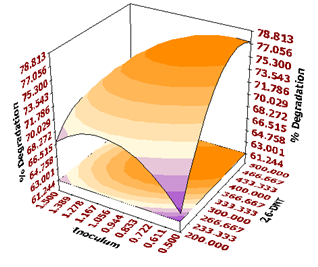
**

**Fig. 5:** 3D surface (left) and 2D contour (right) plot showing the behavior of % biodegradation of 2,6-DNT as a function of MgSO_4_.7H_2_O and temperature.

**
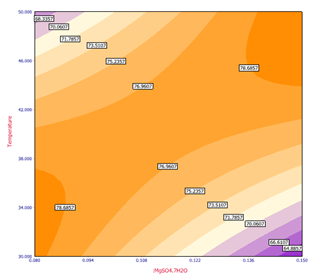
**


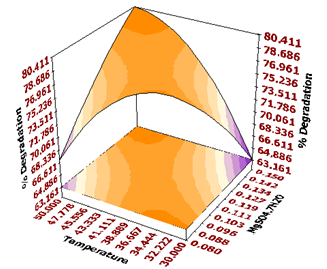


**Table 1.** Factors and their levels in PBD.

| **Variables** | **Abbreviation** | **Units** | **Level 1 (Low)** | **Level 2 (High)** |
| --- | --- | --- | --- | --- |
| 2,4-DNT/2,6-DNT | A | (mg l^-1^) | 100/100 | 400/300 |
| MgSO_4_.7H_2_O | B | (g l^-1^) | 0.05 | 0.1 |
| K_2_HPO_4_ | C | (g l^-1^) | 0.05 | 2 |
| KH_2_PO_4_ | D | (g l^-1^) | 0.05 | 2 |
| Yeast Extract | E | (g l^-1^) | 0.1 | 1 |
| Temperature | F | ^o^C | 15 | 40 |
| pH | G | - | 5 | 9 |
| Agitation | H | rpm | 110 | 200 |
| Inoculum | J | OD | 0.1 | 1 |

**Table 2.** Experimental range and levels of variables used in CCD.

| **Factors** | **- α** | **- 1** | **0** | **+1** | **+ α** |
| --- | --- | --- | --- | --- | --- |
| 2,4-DNT (X_1_)^*^ | 150 | 300 | 450 | 600 | 750 |
| MgSO_4._7H_2_O (X_2_) | 0.045 | 0.08 | 0.115 | 0.15 | 0.185 |
| Temperature (X_3_) | 20 | 30 | 40 | 50 | 60 |
| Inoculum size (X_4_) | 0 | 0.5 | 1 | 1.5 | 2 |

^*^ For 2,6-DNT, - α, -1, 0, +1 and + α levels were 50, 200, 350, 500 and 650 mg l^-1^, respectively

**Table 3 (a).** ANOVA of specific growth rate and biodegradation rate in the screening study of 2,4-DNT biodegradation.


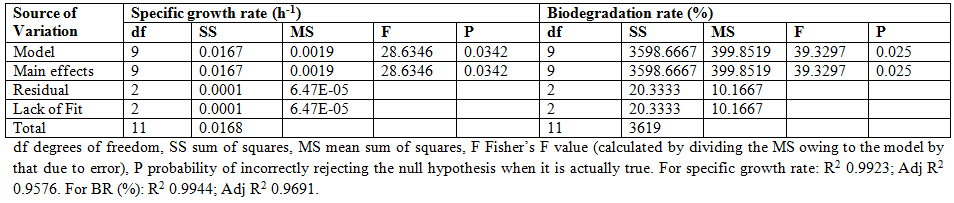


**Table 3 (b).** ANOVA of specific growth rate and biodegradation rate in the screening study of 2,6-DNT biodegradation.


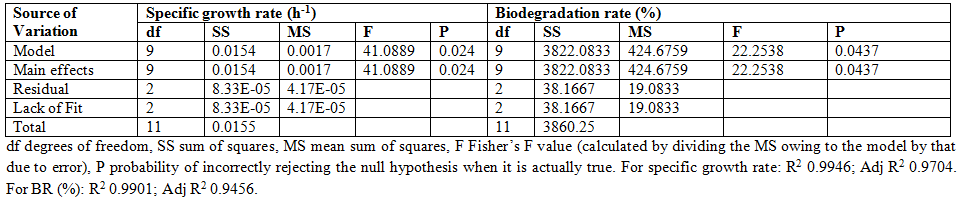


**Table 4 (a).** Regression analysis for Plackett-Burman design variables in 2,4-DNT degradation

**
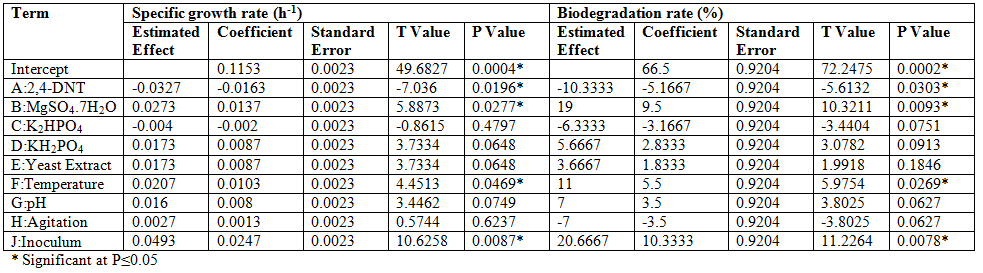
**

**Table 4 (b).** Regression analysis for Plackett-Burman design variables in 2,6-DNT degradation


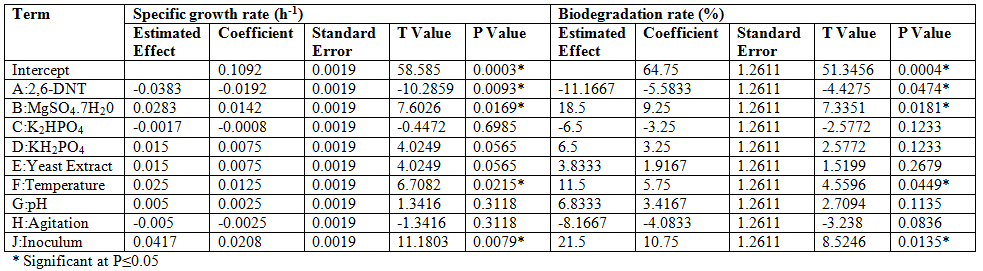


**Table 5.** Comparison of the removal rates of 2,4- and 2,6-DNT reported in literature and in this study

| **Microorganism** | **Maximum concentration (mM) degraded** | **% biodegradation** | **Total biodegradation period** | **Biodegradation**  **(mM h^-1^)** | **Refs.** |
| --- | --- | --- | --- | --- | --- |
| **2,4-DNT** | | | | | |
| *Pseudomonas* sp. | 0.54 | 100% | 120 h | 0.0045 | [1] |
| *Phanerochaete chrysosporium* | 0.25 | 100% | 6 d | 0.0017 | [2] |
| *P. aeruginosa* | 0.25 | 100% | 13 d | 8.01×10^-4^ | [3] |
| *Lactococcus* *lactis* subsp. *lactis* | 0.17 | 100% | 12 h | 0.0141 | [4] |
| *Clostridium acetobutylicum* | 0.54 | 100% | 1 h | 0.54 | [5] |
| *Pseudomonas putida* OU83 | 0.4 | 98% | 48 h | 0.0083 | [6] |
| *Pseudomonas putida* NDT1 | 0.054 | 100% | 15 d | 0.0001 | [7] |
| *S. oneidensis* MR-1 | 0.09 | 100% | 24 h | 0.0037 | [8] |
| *Arthrobacter* sp. K1 | 0.47 | 66% | 10 d | 0.0019 | [9] |
| *S. marisflavi* EP1 | 0.2 | 100% | 24 h | 0.0083 | [10] |
| *R. pyridinivorans* NT2 | 2.6 | 100% | 72 h | 0.036 | Present study |
| **2,6-DNT** | | | | | |
| *Burkholderia cepacia* strain JS850 | 0.1 | 100% | 30 h | 0.0033 | [11] |
| Microbial community | 0.05 | 55% | 32 d | 0.0604 | [12] |
| Microbial community | 0.65 | 100% | 120 h | 0.0054 | [13] |
| *R. pyridinivorans* NT2 | 2.56 | 100% | 72 h | 0.035 | This study |

**References**

1. Spanggord RJ, Spain JC, Nishino SF, Mortelmans KE (1991) Biodegradation of 2,4-dinitrotoluene by a *Pseudomonas* sp. Appl Environ Microbiol 57:3200-05
2. Valli K, Brock BJ, Joshi DK, Gold MH (1992) Degradation of 2,7-dichlorodibenzo-p-dioxin by the lignin-degrading basidiomycete *Phanerochaete chrysosporium*. Appl Environ Microbiol 58(1):221-28
3. Noguera DR, Freedman DL (1996) Reduction and acetylation of 2,4-dinitrotoluene by a *Pseudomonas aeruginosa* strain. Appl Environ Microbiol 62:2257-63
4. Shin K-H, Lim Y, Ahn J-H, Khil J, Cha C-J, Hur H-G (2005) Anaerobic biotransformation of dinitrotoluene isomers by Lactococcus lactis subsp. lactis 27 isolated from earthworm intestine. Chemosphere 61:30-39
5. Hughes JB, Wang CY, and Zhang CL (1999) Anaerobic biotransformation of 2,4-dinitrotoluene and 2,6-dinitrotoluene by *Clostridium acetobutylicum*: a pathway through dihydroxylamino intermediates. Environ Sci Technol 33:1065-1070
6. Walia SK, Ali-Sadat S, Brar R, Chaudhry GR (2002) Identification and mutagenicity of dinitrotoluene metabolites produced by strain *Pseudomonas putida* OU83. Pestic Biochem Physiol 73(2):131-139
7. Hudcova T, Halecky M, Kozliak E, Stiborova M, Paca J (2011) Aerobic degradation of 2,4-dinitrotoluene by individual bacterial strains and defined mixed population in submerged cultures. J Hazard Mater 192:605-613
8. Huang J, Cheng X, Li F, Sheng GD (2013) Reductive biotransformation of 2,4-dinitrotoluene by *Shewanella oneidensis* MR-1 under anaerobic conditions. Microbiol China 40:1734-1741
9. Küce P, Coral G, Kantar Ç (2015) Biodegradation of 2,4-dinitrotoluene (DNT) by *Arthrobacter* sp. K1 isolated from a crude oil contaminated soil. Ann Microbiol 65(1):467-76
10. Huang J, Ning G, Li F, Sheng GD (2015) Biotransformation of 2,4-dinitrotoluene by obligate marine *Shewanella marisflavi* EP1 under anaerobic conditions. Bioresour Technol 180:200-06
11. Nishino SF, Paoli GC, Spain JC (2000) Aerobic degradation of dinitrotoluenes and pathway for bacterial degradation of 2,6-dinitrotoluene. Appl Environ Microbiol 66(5):2139-47
12. Bausum HT, Mitchell WR, Major MA (1992) Biodegradation of 2,4- and 2,6-dinitrotoluene by freshwater microorganisms. J Environ Sci Health A 27(3):663-695
13. Zhang C, Hughes JB, Nishino SF, Spain JC (2000) Slurry-phase biological treatment of 2,4-dinitrotoluene and 2,6-dinitrotoluene: role of bioaugmentation and effects of high dinitrotoluene concentrations. Environ Sci Technol 34:2810-16
